# Sentinel Bleeding as a Sign of Gastroaortic Fistula Formation after Oesophageal Surgery

**DOI:** 10.1155/2014/614312

**Published:** 2014-12-02

**Authors:** M. Uittenbogaart, M. N. Sosef, J. van Bastelaar

**Affiliations:** Department of Surgery, Atrium Medical Centre, Henri Dunantstraat 5, 6419 PC Heerlen, The Netherlands

## Abstract

Gastroaortic fistula formation is a very rare complication following oesophageal resection and, in most cases, leads to sudden death. We report the case of a 65-year-old male with an adenocarcinoma of the oesophagus who underwent neoadjuvant chemoradiation followed by a minimally invasive transthoracic oesophagectomy with gastric tube reconstruction and intrathoracic anastomosis. After an uneventful postoperative course and hospital discharge, the patient reported blood regurgitation on postoperative day 23. Endoscopy revealed an adherent blood clot on the oesophageal wall, which after dislocation caused exsanguination. Autopsy determined the cause of death being massive haemorrhage due to a gastroaortic fistula. The sudden onset of haemorrhage makes this condition particularly difficult to treat. Recognition of warning signs such as thoracic or epigastric pain, regurgitation of blood, or the passing of bloody stools or melena is crucial in the early detection of fistula and may improve patient outcome.

## 1. Introduction

The development of an aortic fistula after oesophagectomy is a life threatening condition mostly due to its lethal nature even when diagnosed at an early stage. This rare complication generally occurs 2-3 weeks after oesophagectomy [1] and often forces surgeons to perform emergency salvage surgery in order to prevent exsanguination. However, in most cases, this diagnosis only becomes apparent at postmortem examination of patients after sudden, unexplained death has occurred [2].

We report a case in which there was in fact a small sentinel or herald bleed, as a prelude to massive exsanguination. Early recognition of a sentinel bleed may be essential to improve patient outcome.

## 2. Case Presentation

A 65-year-old male presented to our outpatient clinic with a three-week history of dysphagia and weight loss. Workup revealed an eT3N3M0 adenocarcinoma of the lower third of the oesophagus. The patient was treated with neoadjuvant chemoradiation, consisting of a five-week course of carboplatin/paclitaxel combined with radiotherapy consisting of 23 fractions of 1.8 Gy, 41.4 Gy in total. Following neoadjuvant therapy a minimally invasive transthoracic oesophagectomy was performed with gastric tube reconstruction with an intrathoracic end-to-side circular stapled anastomosis according to the Ivor-Lewis technique. The postoperative course was uneventful and a postoperative contrast swallow on postoperative day 7 showed no sign of anastomotic leak. The patient was discharged in good condition on the 13th postoperative day.

Histological examination of the resection specimen showed a significant response to the neoadjuvant chemoradiation (tumor regression grade 2 according to Mandard) with a small residual adenocarcinoma invading the submucosa and 11 negative lymph nodes (ypT1N0).

Ten days after hospital discharge, the patient reported regurgitation of small amounts of blood and mild dysphagia. Dietary intake was reduced to fluids only and the patient was scheduled for endoscopy the following day.

Endoscopy of the upper oesophagus and gastric tube showed an intact anastomosis with an adherent blood clot. Dislocation of the clot revealed an uncontrollable massive arterial bleeding and caused severe haemorrhagic shock. Despite endoscopic attempts to control the bleeding and administered advanced life support, the patient succumbed due to exsanguination in the endoscopy suite.

Postmortem examination revealed a 1.5 centimetre diameter dehiscence at the level of the intrathoracic anastomosis with a closely adherent aortic wall, revealing a gastroaortic fistula at the level of the intrathoracic anastomosis without signs of aneurysmal widening of the aortic diameter. The gastrointestinal tract was full of blood, supporting the cause of death being exsanguination due to the gastroaortic fistula. No signs of residual malignancy were found during examination.

## 3. Discussion

Formation of fistulae between the aorta and the gastric tube after oesophagectomy is a very rare phenomenon. The first report of the occurrence of an aortoesophageal fistula was by Dubrueil [3] in 1818, reporting a case of a perforation of the aorta due to ingestion of an avian bone. Ingestion of foreign bodies, most commonly animal bones or dentures, is thought to cause mediastinitis due to pressure necrosis and perforation of the oesophagus, eventually leading to aortitis and fistula formation [4].

### 3.1. Possible Causes of Gastroaortic Fistula

Our case describes a fistula secondary to oesophageal surgery, but in general, aetiology of secondary fistula is diverse, including foreign body perforation, peptic ulceration of the gastric tube, and anastomotic leakage [5]. Most commonly, fistulae are caused by aneurysms of the descending aorta, rupturing into the adjacent oesophagus and thereby causing a fistula between the aorta and the oesophagus [6]. Expansion of the aneurysm increases wall tension, damaging the vasa vasorum of the aorta and thereby gradually weakening the aortic wall. This is thought to cause necrosis of the oesophagus due to the pressure applied on the oesophageal wall. Perforation of the oesophageal wall subsequently causes adherence and inflammation of the aortic wall, resulting in fistula formation [2].

#### 3.1.1. Surgery: Oesophageal Resection and Intrathoracic Stapled Anastomosis

Previous reports have suggested that the degree and method of dissection might predispose for aortic erosion [1]. However, if the cause of massive exsanguination was iatrogenic injury to the vascular wall, one would expect symptoms of blood loss to occur within hours after surgery. In this case, the patient developed symptoms after discharge from the hospital, thus making iatrogenic injury to the vascular wall unlikely the underlying cause of bleeding.

Another possibility is the presence of continuous contact pressure between the linear staples at the level of the anastomosis and the aortic wall causing an erosion of the aorta and eventually resulting in gastroaortic fistula. This mechanism has been previously described as being responsible for formation of tracheogastric fistulae [7]. Thus far there are 23 reports of aortoesophageal fistulae as a complication of oesophagectomy [1]. Up to 60% of patients with a postsurgical gastroaortic fistula develop bleeding between the 9th and 21th days after surgery; 82% occurs between the 2nd and 6th weeks [1].

#### 3.1.2. Neoadjuvant Radiotherapy

**Figure 1:**
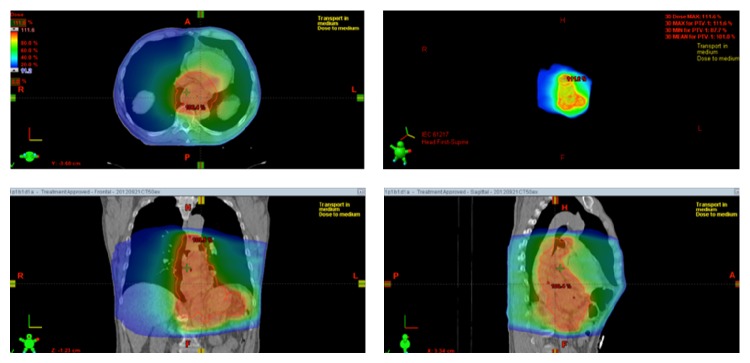
Images of radiation field showing embedding of the anterior wall of the descending aorta and of the oesophagus.

The contribution of neoadjuvant therapy to the formation of fistula is to be considered. Our patient was treated with a five-week course of carboplatin/paclitaxel combined with radiation therapy consisting of 23 fractions of 1.8 Gy (total 41.4 Gy). As shown in Figure 1, along with the oesophagus, the anterior wall of the descending aorta is embedded in the radiation field. It is possible that radiation caused relative ischemia of the oesophagus causing partial destruction of the mucosal barrier. Histological studies have shown that mainly the submucosal layer is affected by the radiation, resulting in teleangiectasia, fibrosis, and neovascularization [8]. Besides causing damage to the epithelium of the intestinal tract, damage to the arterial wall could be expected following radiotherapy. Previous reports have shown the possibility of aortic rupture due to subclinical perivascular infection following radiation therapy [9].

Although this phenomenon is mostly observed six months to five years after radiation therapy [10], it is possible that radiation might contribute to formation of fistulae. Retrospective studies in patients who developed aortaduodenal fistulae and who underwent radiation therapy prior due to malignancy showed that radiation dosis starting at 50 to 60 Gy was associated with chronic intestinal radiation damage and formation of fistula [11].

### 3.2. Diagnosis

The sudden onset of haemorrhage makes this condition particularly difficult to treat. Recognition of warning signs such as thoracic or epigastric pain, regurgitation of blood, or passing of bloody stools or melena is crucial in the early detection of fistula and may improve patient outcome.

Our patient reported dysphagia and regurgitation of blood, in retrospect being the first sign of the underlying pathology. This type of sentinel bleeding has been reported in other types of gastrointestinal surgery, such as pylorus preserving pancreaticoduodenectomy and classic Whipple surgery [12]. Sentinel bleeding as a prelude of an aortoesophageal fistula was first described in the nineteenth century by Chiari-Strassburgh [13], wherein he reported a triad of midthoracic pain or dysphagia, followed by a herald bleed and finally massive hematemesis after a lucid interval. The herald bleed presents mostly 2 to 6 weeks after surgery and the subsequent asymptomatic interval may vary from 30 minutes to 3 days [1]. This symptom-free window of opportunity might be explained by activation of the coagulation cascade due to erosion of the aortic wall, resulting in clot formation, which functions as a plug in the defect. Corrosion by the gastrointestinal contents or even bacterial activity might weaken the clot, as such causing the catastrophic bleed [1].

Although early recognition of this sequence of events seems essential in order to maximize the survival, there is no apparent consensus on the optimal diagnostic strategy. Endoscopy of the upper gastrointestinal tract might aid in the detection of fistula. Endoscopic findings may be directly due to visualization of pulsatile blood into the oesophagus or a pulsatile adherent blood clot. More subtle findings during endoscopy might point in direction of a fistula, such as submucosal hematoma shown as a blue-gray discoloration of the oesophageal wall [5]. However, the sensitivity of detection of fistulae via endoscopy is a mere 38% [14]; therefore endoscopy might be of more use to exclude other common causes of upper gastrointestinal bleeding, such as Mallory-Weiss lesions and peptic ulcers [1]. Arterial contrast studies, such as aortography or even computed tomography scans with arterial contrast, are potentially useful in the detection of fistula, but during the symptom-free interval radiological evidence of the fistula may be absent due to the transient clot formation [15].

### 3.3. Treatment

The literature reports twenty-three cases of gastroaortic fistula following oesophageal surgery, all but three reporting a fatal outcome despite surgical efforts [1]. The main problem is the delay in diagnosis resulting in a limited time frame in which salvage surgery can be performed; the majority of patients will perish before reaching the operating theatre. Therefore the solution has to be sought in temporary measures to prolong the interval between diagnosis and exsanguination. Use of the Sengstaken-Blakemore tube was first described in 1966 as being a successful as a bridge to definite surgical repair [16], but the much disputed pressure necrosis of the oesophageal wall [17] has caused this device to become obsolete. With the ongoing advancements in endovascular as well as endoscopic techniques, stenting the aorta or even oesophagus might be helpful in case of haemorrhagic shock, functioning as a bridge to definitive and major surgical repair [18].

## 4. Conclusion

Patients reporting regurgitation of blood or hematemesis following an oesophagectomy should raise suspicion of a gastroaortic fistula. This sentinel or herald bleed should prompt us to actively “seek the leak.” It is essential to recognise the lucid interval between the sentinel bleed and the massive hematemesis and utilize it to rapidly confirm diagnosis and create a treatment plan. Depending on the extent of the bleeding, we would advise to start with a computed tomography scan with arterial contrast to visualise the origin of the bleeding and determine the best surgical approach, either endovascular, endoscopic, or invasive salvage surgery. In case of an inconclusive scan, a subsequent endoscopy of the upper gastrointestinal tract might confirm the suspicion. However, despite early diagnosis and extensive surgical effort, reported patient outcome is poor.

## Learning Points

(i) Regurgitation of blood or hematemesis should be recognised as a possible sentinel bleed due to a gastroaortic fistula. (ii) Sentinel or herald bleed after oesophagectomy should prompt us to actively “seek the leak” and treat it. (iii) Computed tomography scanning with arterial contrast or endoscopy of the upper gastrointestinal tract should be used to confirm diagnosis. (iv) Despite early diagnosis and surgical effort, patient outcome is poor.

## References

[1] Molina-Navarro C., Hosking S. W., Hayward S. J., Flowerdew A. D. S. (2001). Gastroaortic fistula as an early complication of esophagectomy. *The Annals of Thoracic Surgery*.

[2] Byard R. W. (2013). Lethal aorto-oesophageal fistula—characteristic features and aetiology. *Journal of Forensic and Legal Medicine*.

[3] Dubrueil (1818). Observations sur la perforation de l'oesophage et de l'aorte thoracique par une potion d'os avale: avec des reflexions. *J Univ Sci Med*.

[4] Amin S., Luketich J., Wald A. (1998). Aortoesophageal fistula: case report and review of the literature. *Digestive Diseases and Sciences*.

[5] Heckstall R. L., Hollander J. E. (1998). Aortoesophageal fistula: recognition and diagnosis in the emergency department. *Annals of Emergency Medicine*.

[6] Hollander J. E., Quick G. (1991). Aortoesophageal fistula: a comprehensive review of the literature. *The American Journal of Medicine*.

[7] Chen Y. Y., Chang J. M., Lai W. W. (2012). Tracheo-neo-esophageal fistula caused by exposed metallic staples erosion. *Annals of Thoracic Surgery*.

[8] Drognitz O., Pfeiffenberger J., Schareck W. (2002). Primäre aortoduodenale Fistel als Spätkomplikation nach paraaortaler Radiatio Ein Fallbericht. *Der Chirurg*.

[9] McCready R. A., Hyde G. L., Bivins B. A., Mattingly S. S., Griffen W. O. (1983). Radiation-induced arterial injuries. *Surgery*.

[10] Galland R. B., Spencer J. (1986). Radiation-induced gastrointestinal fistulae. *Annals of the Royal College of Surgeons of England*.

[11] Berthrong M., Fajardo L. F. (1981). Radiation injury in surgical pathology. Part II. Alimentary tract. *The American Journal of Surgical Pathology*.

[12] Yekebas E. F., Wolfram L., Cataldegirmen G., Habermann C. R., Bogoevski D., Koenig A. M., Kaifi J., Schurr P. G., Bubenheim M., Nolte-Ernsting C., Adam G., Izbicki J. R. (2007). Postpancreatectomy hemorrhage: diagnosis and treatment—an analysis in 1669 consecutive pancreatic resections. *Annals of Surgery*.

[13] Chiari-Strassburgh H. (1914). Injury of the esophagus with perforation of the aorta produced by a foreign body (Ueber Fremdkorperverletzung des Oesophagus mit Aortenperforation). *Berliner klinische Wochenschrift*.

[14] Perdue G. D., Smith R. B., Ansley J. D., Costantino M. J. (1980). Impending aortoenteric hemorrhage: the effect of early recognition on improved outcome. *Annals of Surgery*.

[15] Khawaja F. I., Varindani M. K. (1987). Aortoesophageal fistula: review of clinical, radiographic, and endoscopic features. *Journal of Clinical Gastroenterology*.

[16] Valtonen E. J., Koivuniemi A. (1967). Aortoesophageal fistula complicating carcinoma of the esophagus. Report of observations in two cases. *Journal of Thoracic and Cardiovascular Surgery*.

[17] Vlavianos P., Gimson A. E. S., Westaby D., Williams R. (1989). Balloon tamponade in variceal bleeding: use and misuse. *British Medical Journal*.

[18] Okita Y., Yamanaka K., Okada K. (2014). Strategies for the treatment of aorto-esophageal fistula. *European Journal Cardio-Thoracic Surgery*.

